# Intraplacental Choriocarcinoma: Rare or Underdiagnosed? Report of 2 Cases Diagnosed after an Incomplete Miscarriage and a Preterm Spontaneous Vaginal Delivery

**DOI:** 10.1155/2017/7892980

**Published:** 2017-04-16

**Authors:** Rita Ferraz Caldas, Paula Oliveira, Cátia Rodrigues, Inês Reis, Horácio Scigliano, Rosete Nogueira, Célia Araújo, Soledade Ferreira

**Affiliations:** ^1^Department of Obstetrics and Gynecology, Centro Hospitalar de Entre o Douro e Vouga, Santa Maria da Feira, Portugal; ^2^Department of Anatomic Pathology Lab Albino Oliveira, Centro Hospitalar de Entre o Douro e Vouga, Santa Maria da Feira, Portugal; ^3^Department of Anatomic, Cytopathology and Thanatology, Escola Superior de Saúde, Instituto Politécnico do Porto, Porto, Portugal; ^4^Department of Fetal Pathology and Anatomic Pathology Lab, CGC Genetics, Porto, Portugal; ^5^Life and Health Sciences Research Institute (ICVS), School of Medicine, ICVS/3B's-PT Government Associate Laboratory, Campus of Gualtar, University of Minho, Guimarães, Braga, Portugal

## Abstract

Intraplacental choriocarcinoma is a rare malignant tumor diagnosed after an abortion, an ectopic pregnancy, or a term or preterm pregnancy or following the diagnosis of a hydatidiform mole. During pregnancy, it may be more common than reported, as most patients are asymptomatic and placental choriocarcinomas are usually inconspicuous macroscopically and are often mistaken for an infarct. Based upon a case study methodology, we describe 2 cases of intraplacental choriocarcinoma: the first case was identified in the product of a uterine curettage following an incomplete miscarriage and the second in one of the placentas of a bichorionic twin pregnancy. Maternal investigation did not reveal evidence of metastatic disease and neither did the infants' one in the second case. The two cases underwent maternal surveillance with serum hCG and remained disease-free until the present. In conclusion, intraplacental choriocarcinoma is easily underdiagnosed but with current treatment, even in the presence of metastasis, the prognosis is excellent. A routine microscopic examination of all the placentas and products of miscarriage can increase the real incidence of this entity and consequently improve its management.

## 1. Introduction

The gestational trophoblastic disease includes a spectrum of tumors from hydatidiform moles to more malignant forms: the invasive mole, the placental site, and epithelioid trophoblastic tumors and choriocarcinoma. Intraplacental choriocarcinoma is a focal neoplastic proliferation of the chorionic villous trophoblast. It is a rare variant of gestational choriocarcinoma but, due to its rareness, the information available is still limited, with scarce data derived from individual case reports or small case series (only 62 cases reported in the literature [1]). It accounts for no more than approximately 0.04% of all gestational trophoblastic diseases [2–4].

However, as the placenta's histological examination is not routinely performed and the lesions have an inconspicuous appearance, the real incidence is probably underestimated [3]. In some case reports, the diagnosis followed the first trimester abortions, in others it followed hydatidiform moles and preterm or term pregnancies [1, 5, 6], with most cases identified in the third trimester in asymptomatic women [4].

The aetiology, pathogenesis, natural history, and adequate therapy of choriocarcinoma are still unknown. The reported cases in the literature describe different outcomes. In some cases, the authors describe fatal outcomes associated with maternal or fetal/infant metastatic disease (particularly to the lungs) [6, 7], while in others there were no implications for the mother and infant [8]. Complications as intrauterine growth restriction, premature birth, fetal anaemia, and transplacental haemorrhage have also been reported [1–3].

Because this disease is potentially curable and a longer interval between delivery and treatment adversely affects the outcome, the early diagnosis is important [7–10].

Treatment trends have also been changing. For metastatic disease, the prognosis was very poor before 1980, with the 5 cases reported resulting in maternal death. A few authors described that the administration of methotrexate alone has been successful in treating metastases [9, 11]. Hysterectomy without adjuvant treatment showed to be ineffective but after starting the treatment with chemotherapy the prognosis improved dramatically, with a recently reported long term remission rate of 100% [1]. In nonmetastatic disease, isolated maternal serum hCG surveillance was recommended in a recently published management guidance [1]. Additional surgery or chemotherapy seems to be unnecessary.

The objective of this article is to describe 2 cases of intraplacental choriocarcinoma diagnosed after a clinical suspicion of an incomplete miscarriage and a preterm spontaneous vaginal delivery. We reviewed the clinical files of the mothers diagnosed with intraplacental choriocarcinoma and of their infants and the study was approved by the Local Research Ethics Committee.

## 2. Case Presentation

### 2.1. Case 1

Primigravida, 29 years old, with no significant obstetric or medical history, presented with vaginal bleeding and an 8-week amenorrhea in December 2010.

**Figure 1:**
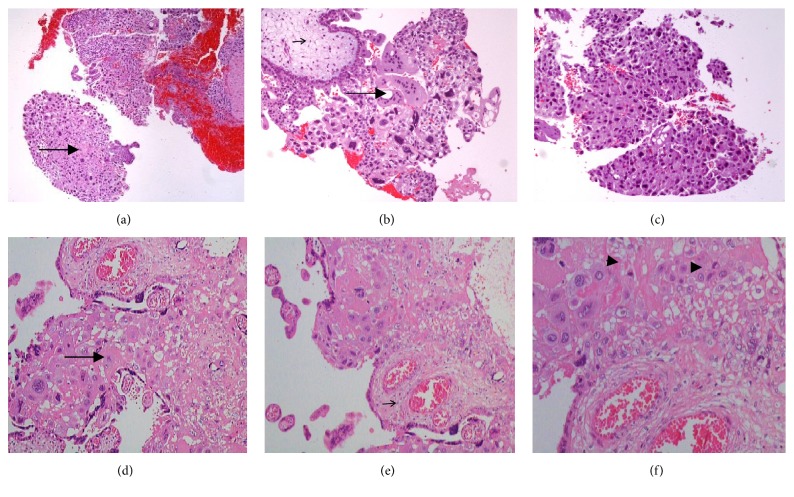
Placental histological features from case 1 at 8 weeks of gestational age [(a), (b), and (c)] and case 2 at 32 weeks of gestational age [(d), (e), and (f)]. Chorionic villi (small arrow) surrounded by tumor trophoblast cells [cytotrophoblast and intermediate trophoblast with patchy small foci of syncytiotrophoblast (arrow)]. Mitotic figures (head arrow).

Physical examination was normal. The ultrasound showed a placenta with hypoechogenic vesicular formations with no embryo. The serum hCG level was 141723 mUI/mL. An aspirative curettage was performed. On microscopic examination, slid sheets of cytotrophoblastic and multinucleated syncytium without stroma were identified (Figures 1(a), 1(b), and 1(c)).

The chest CT scan and the brain MRI showed no metastasis. One month after the curettage, the serum hCG levels spontaneously returned to normal. The follow-up in the next 2 years, without therapy, was negative.

In December 2012, she had a subsequent pregnancy, complicated by gestational diabetes. Labour was induced at 39 weeks and she delivered vaginally a 3470 g female newborn. The placenta's histopathological examination had no signs of intraplacental choriocarcinoma and the serum hCG levels were negative after birth. The infant had no signs of metastatic disease and remains well.

### 2.2. Case 2

Primigravida, 29 years old, with no significant obstetric or medical history, had an uncomplicated pregnancy with normal fetal ultrasounds throughout the gestation. She spontaneously delivered vaginally, bichorionic twins at 32 weeks and 5 days, in August 2013. The newborns weighed 2020 g and 1935 g. They were admitted in the NICU due to prematurity. The postpartum period was uneventful. The placentas (fused) had no macroscopically visible lesions but were sent to routine histological examination due to the preterm delivery.

The placenta corresponding to the first twin was 19 × 10 × 3 cm and the microscopic examination showed an intraplacental choriocarcinoma characterized by focal proliferation of both cytotrophoblast and syncytiotrophoblast in the intervillous space without involvement of the adjacent chorionic villi (Figures 1(d), 1(e), and 1(f)). There was no invasion of the stromal or vascular tissues. The maternal physical examination, ultrasound, serum hCG levels, chest CT scan, and brain MRI were normal one month after the delivery.

The follow-up is negative until the present with only hCG surveillance. Both mother and infants have been disease-free for 3 years.

## 3. Discussion

Intraplacental choriocarcinoma is a rarely reported malignant tumor that arises from chorionic villous trophoblast. The majority of the cases reported in the literature follow the diagnosis of a hydatidiform mole [1, 2], but they can follow an otherwise normal nonmolar placenta. The first report was in 1963 when Driscoll described an “incidental finding of a choriocarcinoma within a term placenta” [12]. Fukunaga and Nomura also defined it as a “neoplastic trophoblast proliferation localized on the placenta that implies the absence of metastases” [8]. However, other authors described associated maternal or fetal metastatic lesions, particularly to the lungs, that may be fatal to both. Occasionally, a tumor in the placenta is not identified despite metastasis in the neonate. In some of these cases, this was certainly due to the lack of placental examination. Massive transplacental fetal haemorrhage has also been reported [3, 5, 6, 9, 10]. Thus, some authors consider it more accurate to use the term “intraplacental choriocarcinoma” because of this possibility of distant metastases.

The prevalence of choriocarcinoma is low but it is most often seen in women aged less than 15 or more than 45 years old, as well as in multiparous women [2, 3, 5]. However, due to the subtle nature of the gross appearance of these lesions, it is likely that many cases go undiagnosed and the real incidence is higher than documented.

The aetiology and pathogenesis of the lesions are still unknown [1–5, 10, 11].

Prenatal diagnosis of this entity is rarely possible and inconsistent but a mass lesion can be found. The maternal symptoms, when present, are also nonspecific. The most common presentation is the vaginal bleeding but the symptoms related to metastasis like haemoptysis or cough have been reported [1, 3, 11]. However, in the majority of the cases, the disease is clinically silent and the diagnosis is based on the pathological placental or RPOC examination. Fetal and obstetric reported complications include fetomaternal haemorrhage, placental abruption, fetal hydrops, anaemia, intrauterine growth restriction, and fetal death [1, 2, 5, 9, 12]. In our first case, vaginal bleeding was the symptom that led the woman to the hospital. The second case was diagnosed incidentally in one of the placentas of a twin pregnancy when the placenta was examined due to a preterm delivery. The mother had no symptoms and there were no fetal complications related to the tumor.

Regarding the pathological findings, usually the lesions are not macroscopically visible or have an inconspicuous appearance, being often mistaken for an infarct [6, 8, 9]. Liu and Guo reviewed the different gross appearance of the lesions and identified single or multiple nodules similar to infarctions [13]. Microscopically, the focal proliferation of both cyto- and syncytiotrophoblast (biphasic) in the stem villi that project to the intervillous space, with significant necrosis in the centre, is the typical pattern. Some reports describe stromal or vascular invasion [6, 8, 9]. In both our cases, there was microscopic proliferation of both cytotrophoblast and syncytiotrophoblast, occupying the intervillous space with no stromal or vascular invasion.

Detailed maternal physical examination, serum hCG, X-ray, ultrasound, CT scan, and MRI should always be performed to rule out metastasis when a choriocarcinoma in situ is suspected or confirmed because clinical outcomes appear to depend on the presence of metastasis [1, 2, 5, 7, 9, 10, 13, 14].

The optimal treatment of intraplacental choriocarcinoma is still unclear. According to one review in 2003, before chemotherapy was available, the overall survival at 5 years for choriocarcinoma with hysterectomy alone was 41% in the absence of metastases and 19% in the metastatic cases. Chemotherapy increased survival to almost 100% [9]. However, treatment of nonmetastatic disease is controversial. Some authors describe cases of success with only surveillance [1, 3, 8, 14, 15]. Some authors suggested that “patients with choriocarcinoma confined to the placenta with rapid decrease of serum hCG after the removal of the placenta do not need chemotherapy, but only fetal and maternal close follow-up” [16]. In a recent review and management guidance published in 2016, only surveillance in cases of nonmetastatic disease is also recommended [1]. In our cases, there were no signs of dissemination and serum hCG decreased rapidly, so the women were not treated with chemotherapy and were kept under surveillance with beta-HCG and imaging, with negative follow-up until now. In the first case, the woman was pregnant after 3 years and had no complications or signs of the disease.

In conclusion, intraplacental choriocarcinoma may manifest as a spectrum of clinical entities ranging from an incidental lesion diagnosed on the pathological examination of the placenta, with no adverse effects on mother or infant, to a metastatic maternal or infantile disease. Due to the potential fatal outcome of this entity if not treated [4, 6, 17, 18], careful evaluation of both mother and infant after the diagnosis is important. The real prevalence of the disease may actually be higher than documented since it is not routine practice to analyse all the placentas following each delivery. As the early diagnosis and appropriate management are essential, the health providers should have an increased awareness of the existence of these lesions and its manifestations.
